# Editorial: Multidimensionally decoding the impact of tumor heterogeneity on immunotherapy responsiveness in gastrointestinal tumors

**DOI:** 10.3389/fimmu.2024.1482206

**Published:** 2024-09-03

**Authors:** Ruizhi Wang, Libo Wang, Yuqing Ren, Xinwei Han, Zaoqu Liu

**Affiliations:** ^1^ Department of Interventional Radiology, The First Affiliated Hospital of Zhengzhou University, Zhengzhou, Henan, China; ^2^ Department of Pancreatic Cancer, Tianjin Medical University Cancer Institute and Hospital, National Clinical Research Center for Cancer, Key Laboratory of Cancer Prevention and Therapy, Tianjin, China; ^3^ Department of Respiratory and Critical Care Medicine, The First Affiliated Hospital of Zhengzhou University, Zhengzhou, Henan, China; ^4^ Interventional Treatment and Clinical Research Center of Henan Province, Zhengzhou, Henan, China; ^5^ Interventional Institute of Zhengzhou University, Zhengzhou, Henan, China; ^6^ Institute of Basic Medical Sciences, Chinese Academy of Medical Sciences and Peking Union Medical College, Beijing, China

**Keywords:** immunity, multidimension analysis, tumor heterogeneity (TH), immunotherapy responsiveness, gastrointestinal tumors

Recent advances in cancer biology and immunobiology have revolutionized medical oncology, positioning cancer immunotherapy as a burgeoning treatment modality. This new generation therapy, which follows traditional interventions like surgery, radiotherapy, and chemotherapy, modulates the immune system to reinitiate and sustain the “tumor-immunity” cycle. It enhances the body’s anti-tumor immune response by boosting the recognition and eradication of tumor cells, thereby potentially controlling or specifically eliminating tumors (1). However, the response to immunotherapeutic agents varies among patients; only a minority achieve significant clinical benefits (2). This variability primarily stems from tumor heterogeneity, influenced by the distinct tumor microenvironments (3).

Decoding cellular heterogeneity within the tumor microenvironment and elucidating spatial architectures are crucial for understanding disease progression and therapeutic responses. These insights enable the precise tailoring of treatment regimens in clinical settings, including the selection of patient subsets for immunotherapy and the appropriate reassignment of other subsets. A suite of advanced technologies, such as single-cell sequencing, spatial transcriptomics, liquid and tissue biopsies and artificial intelligence (AI), facilitates this process. AI integrates multi-omics data layers, including genomics, transcriptomics, proteomics, epigenomics, and metabolomics, enhancing the assessment of tumor heterogeneity and the efficacy of immunotherapy (4, 5). This comprehensive analysis aids in unraveling the molecular underpinnings of tumor immunotherapy, systematically categorizing tumor subtypes, identifying specific biomarkers efficiently, predicting clinical outcomes accurately, and developing personalized treatment strategies (6).

**Figure 1:**
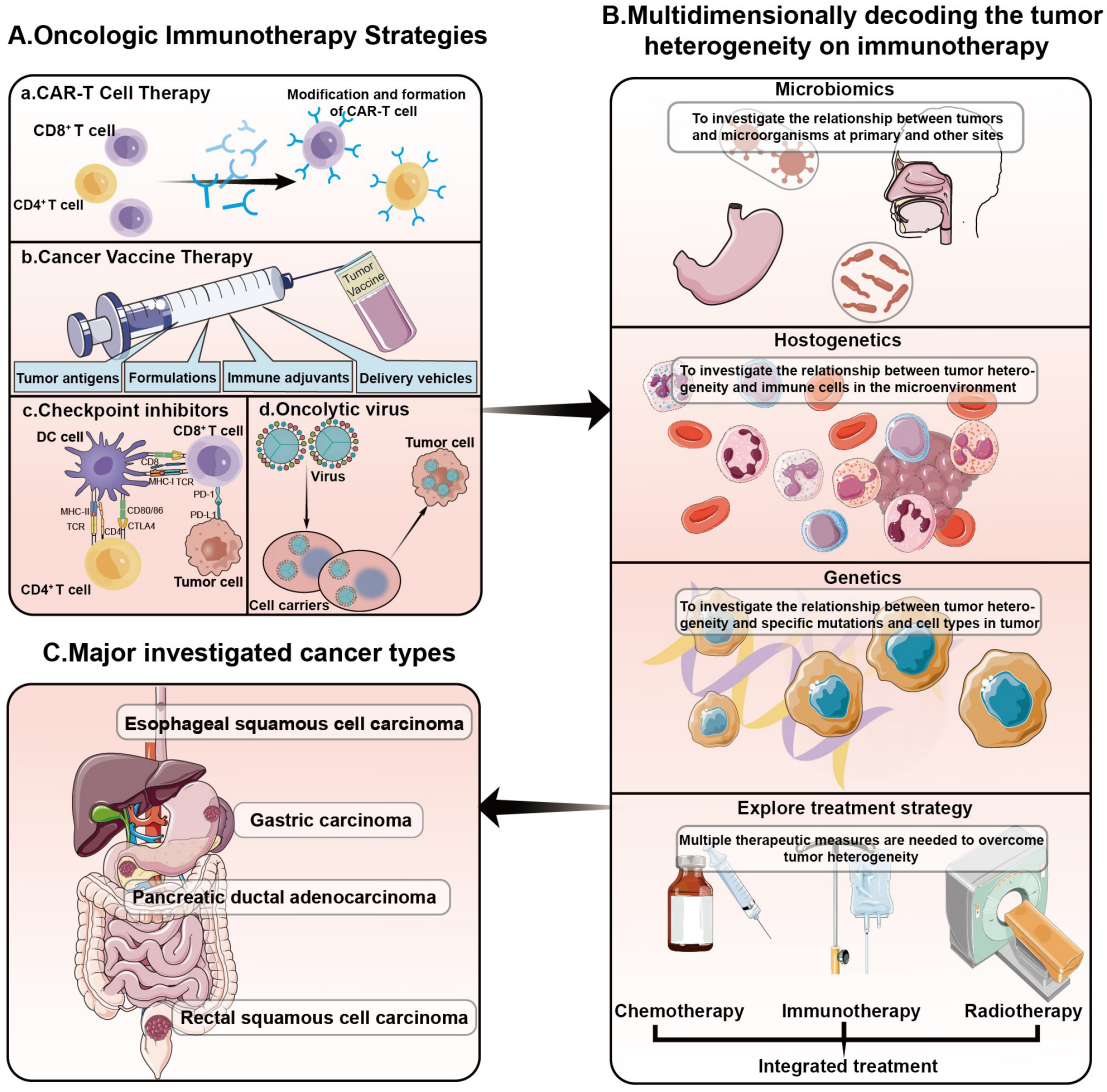
Decoding the impact of tumor heterogeneity on immunotherapy in a variety of gastrointestinal tumors. **(A)** Oncologic immunotherapy strategies. At present, the commonly used methods of cancer immunotherapy in clinical practice include: CAR-T cell therapy, cancer vaccines, immune checkpoint inhibitors, oncolytic viruses and so on. **(B)** Common exploration of tumor heterogeneity includes heterogeneity of the microbiome, heterogeneity of tumor immunity, and heterogeneity of tumor genomics. Combined with these heterogeneous conditions, the use of chemotherapy, radiotherapy, immunotherapy and other therapies can be further explored. **(C)** The tumor types we explored in this Research Topic are shown in the figure: esophageal squamous cell carcinoma, gastric carcinoma, pancreatic ductal adenocarcinoma, rectal squamous cell carcinoma.

In recent years, the prevalence of gastrointestinal tumors has increased, yet their prognosis remains largely unchanged. Tumor heterogeneity has been identified as a principal factor in this stagnation. Extensive research has addressed the heterogeneity of gastrointestinal tumors, encompassing esophageal, colorectal, hepatobiliary, and pancreatic cancers, and has explored targeted precision therapies (7–10). Our research aims to deepen the understanding of tumor heterogeneity within the realm of gastrointestinal cancer, thereby enhancing the multidimensional comprehension of immunotherapy. Our approach spans several dimensions: developing biomarkers for pre-treatment assessment, identifying populations sensitive to immunotherapy at early stages, devising new combination treatment strategies, and investigating the factors, underlying mechanisms, and potential interventions for immunotherapy-nonresponsive patients. This comprehensive analysis is poised to revolutionize the development of personalized immunotherapeutic strategies by elucidating the impact of tumor heterogeneity on treatment responsiveness (**Figure 1**).

This Research Topic compiles contributions from 44 authors across five articles, exploring the crucial role of decoding tumor heterogeneity in tumor immunity and the responsiveness to immunotherapy in gastrointestinal cancers. Qu et al. reported on a rare case of rectal squamous cell carcinoma (RSCC), which represents a small fraction of rectal malignancies. The advent of immunotherapy has opened new avenues for treating malignant tumors; however, its application in RSCC, particularly in cases with high PD-L1 expression, remains unexplored. This article details the treatment journey of an RSCC patient, revealing that single-agent immune checkpoint inhibitors (ICIs) may be less effective than combined strategies incorporating radiotherapy. Similar findings have been observed in advanced esophageal squamous cell carcinoma. Huang et al. evaluated the efficacy of ICIs combined with concurrent chemoradiotherapy (CCRT) versus CCRT alone in a retrospective cohort study involving patients with locally advanced esophageal squamous cell carcinoma. Preliminary data from single-center studies indicate that adding ICIs to CCRT does not enhance survival outcomes compared to CCRT alone. Moreover, gastric cancer exhibits significant heterogeneity, with microbiomics emerging as a pivotal research direction. Munoz-Medel et al. proposed that P. gingivalis and its virulence factors may serve as mechanistic links between oral health and the onset and progression of gastric cancer. They explored the influence of bacterial virulence on the efficacy of inflammatory responses and tumor immune checkpoint inhibitors, offering innovative avenues for therapeutic interventions. Further addressing tumor heterogeneity, Wang et al. utilized Mendelian randomization to elucidate the relationship between immune cell infiltration and gastrointestinal cancers at various sites. This study opens new avenues for devising prevention strategies and identifying novel immunotherapeutic targets. Additionally, Ye et al. discovered a unique proliferating cell type, termed Prol, characterized by an abundance of cell cycle and mitotic genes, using single-cell RNA sequencing and spatial transcriptome technology. In pancreatic ductal adenocarcinoma (PDAC), a higher proportion of Prol cells correlates with reduced overall survival (OS) and progression-free survival (PFS). Conversely, a lower proportion of these cells appears to enhance the efficacy of immunotherapy and gemcitabine.

This topic aggregates five manuscripts that explore the critical intersections of tumor heterogeneity and immunotherapy responsiveness in gastrointestinal cancers. These studies synergistically merge diverse data from various research methodologies, enhancing our understanding of cancer subtype heterogeneity. They successfully identify key immune biomarkers specific to gastrointestinal tumors and underscore the significant potential of leveraging tumor heterogeneity in the immunotherapeutic treatment of these cancers. Collectively, these insights contribute profoundly to the development of precision medicine modalities, paving the way for tailored therapeutic strategies.
